# Supplementary material for: Patterns of Intron Gain and Loss in Fungi
Source: PLoS Biol. 2004 Nov 30;2(12):e422. doi: 10.1371/journal.pbio.0020422 (PMC532390; doi:10.1371/journal.pbio.0020422)
Supplement: Table S1 — Also available at http://genes.mit.edu/NielsenEtAl/. (4.3 MB ZIP). [file pbio.0020422.st001.zip › NielsenEtAl/html/465.html]

AN1069.1.NCU05720.1.MG03122.1.FG02729.1


```
 CLUSTAL W (1.82) Multiple Sequence Alignments - Introns Inserted


Sequence 1: MG03122.1	332 aa
Sequence 2: FG02729.1	334 aa
Sequence 3: NCU05720.1	358 aa
Sequence 4: AN1069.1	331 aa
Alignment Length: 359 aa
Number Identitical Residues: 178 aa
Alignment Score (without introns) 8932


MG03122.1 	MPGFGDFTSLCQMAPLPLCSNVGKVNNIIG---IEPNCYARSIELANTIIFQGAASAMHI
NCU05720.1	MGKFGDFSSICRMAPIPLCASVGPITSIATGVGIEPDCYARNIEVANTIIFQGAASVMHI
FG02729.1 	MSGFGDFTTICETAPLPLCAAVGPVLQATGRTGIEPECYARNIELANTIIFEGATAVMHI
AN1069.1  	-MGFGDFDEICKKAALPLCSLVGPSSAISGSTGIIPNCYARNIELANTIIFEGAASFLHI
          	   ****  :*. *.:***: **         .* *:****.**:******:**:: :**

MG03122.1 	VALCMTVVMVLHVRGKFTAVG1RKEILNFFYFYMLLTFISLCVDAGVVPPGSGPYPYFVS
NCU05720.1	VALVMTVVMLLHVRGKFTAVG1RKEITTFFYLYMILTFLSLCIDAGVIPPHSGSYPYFVA
FG02729.1 	VALVMTVIMILHVRSKFTAVG~RKEILSFFYLYMLLSAMSLVIDAGVVPPGSDPYPYLVS
AN1069.1  	IALGMTVIMILHIRSKFTAVG1RKEIITFFYIYMALTVCSLVIDAGVVPPRSGPFPWFVA
          	:** ***:*:**:*.****** **** .***:** *:  ** :****:** *..:*::*:

MG03122.1 	VQNGLTSSLVTCLLINGFVGFQLYEDGTPLSLWMMRICSIVAFAITFLVSLATFKSWVGL
NCU05720.1	VQAGLASALVTCLVINGFVGFQLYEDGTPLSLWMLRLCSFVAFVISFLVGLATFKSWAGL
FG02729.1 	VQNGFSSAVITCLLINGFVGFQLYEDGTPLSVWMLRISTLAAFTISFLVSLATFKSWAGL
AN1069.1  	VQNGLTSALCTSLLVNGFVGFQLYEDGTALSVWLLRLTSTVMFAVSFLISILTFKSWGGL
          	** *::*:: *.*::*************.**:*::*: : . *.::**:.: ***** **

MG03122.1 	SPSNTIGLFVVLYLLNAVQLFVYVVLQVMLVVRTLHERWPLGDIAFGVFFFVLGQVILYA
NCU05720.1	GPTNTVGIFVVLYFLNALQLLLYVVMQIILVTRTLQDRWPLGDIAFGLFFFIAGQVILYA
FG02729.1 	SPTNTVGLFVVLYLLNAIQLFIYVAMQILLVTRTLHDRWPLGDIAFGIFFFVAGQVFLYA
AN1069.1  	SPTNTLAMFIVLYIINAICIAVYLVMQLLLVLNTLEDRWPLGHIAFGLLVFIAGQVIMYA
          	.*:**:.:*:***::**: : :*:.:*::** .**.:*****.****::.*: ***::**

MG03122.1 	LSTPICEAVSHYMDGLFFATTCNLLGVMMVYK0YWDSITKEDLEFSVGTRMNNWEVKELL
NCU05720.1	FSSPICEGISHYLDGLFFATTCNLLAVMMVYK~YWDSITKEDLEFSVGTRMNNWEVKELL
FG02729.1 	FSSKICNAISHYLDGLFLATVCNLLGVMMVYK0YWDSITKEDLEFSVGTRMNNWEVKELL
AN1069.1  	FGDVICDNVQHYLDGLFFATFCNLLAVMMVYK0FWDYITKEDLEFSVGIKPNNWEIKELL
          	:.  **: :.**:****:** ****.****** :** *********** : ****:****

MG03122.1 	P---EEDRRQTIYTEDPYG-------------QSSGYD--------HPYSPNTAARPYSK
NCU05720.1	PQGPEEDRRATVYADPIYEGHYASGPGTGSGASASGYEGGHHRRESHGYTPS-PNRQSLR
FG02729.1 	P---EEERRATVFSEDLYG-------------QNSSYD--------LPYSPG-AARYSAK
AN1069.1  	P---DEDRRTTVYQD-----------------TNSEYAG-------SMYHHRASTYNGHG
          	*   :*:** *:: :                   * * .         *   :.      

MG03122.1 	Y
NCU05720.1	Y
FG02729.1 	Y
AN1069.1  	Y
          	*
```
